# Epidemiological features and outcomes of patients with psoas abscess: A retrospective cohort study

**DOI:** 10.1016/j.amsu.2021.01.040

**Published:** 2021-01-18

**Authors:** Takeaki Sato, Daisuke Kudo, Shigeki Kushimoto

**Affiliations:** Department of Emergency and Critical Care Medicine, Tohoku University Hospital, 1-1 Seiryo-machi, Aoba-ku, Sendai, 980-8574, Japan

**Keywords:** Psoas abscess, Secondary abscess, Pyogenic spondylitis, *Staphylococcus aureus*, Epidemiology

## Abstract

**Background:**

Psoas abscess (PA) is an uncommon disease. Although PA is associated with significant morbidity and mortality, its epidemiology and clinical characteristics remain unknown. This study aimed to evaluate the epidemiological and clinical features and outcomes of patients with PA in a prefectural-wide study.

**Materials and methods:**

This was a multicenter retrospective cohort study conducted between 2010 and 2012 in the Miyagi prefecture with a population of 2,344,062 in 2011. Adult patients with PA were enrolled from 71 secondary and tertiary care hospitals.

**Results:**

There were 57 patients with adult PA in the Miyagi prefecture. The median age of the patients was 72 years, and 67% patients were male. Fever and flank pain were the primary symptoms in 82% and 74% of patients, respectively. Ten patients (18%) had septic shock, and the hospital mortality rate was 12%. Secondary PA was present in 72% of cases, and the most common origin was pyogenic spondylitis. Of the patients with secondary PA, 44% had an epidural abscess. The most common pathogens were *Staphylococcus aureus*, and 11% (6 cases) of the cases were caused by methicillin-resistant *S. aureus*.

**Conclusion:**

In the Miyagi prefecture of Japan, the estimated prevalence of PA was 1.21/100,000 population years and hospital mortality was 12%. Secondary PA accounted for more than 70% of the cases, and *S. aureus* was the most common causative pathogen.

## Highlights

• The hospital mortality rate of psoas abscess (PA) was 12% in Japan.
• Estimated prevalence of PA was 1.21/10,000 population years between 2010 and 2012.
• Secondary PA: >70% of cases with the most common origin as pyogenic spondylitis.
• Epidural abscess formed in 44% of patients.
• The most common pathogen was *Staphylococcus aureus*.

## 1 Introduction

Psoas abscess (PA) is an uncommon disorder that is often missed by physicians owing to its nonspecific clinical presentation [1]. PA can lead to significant morbidity and mortality, with a reported mortality rate of 5–15% and relapse rate of 15.8% [1,2].

Computed tomography (CT) is considered the optimal diagnostic modality for evaluating PA. There are limited epidemiological studies on patients with PA. In the era of the first generation of CT, a report from UK showed the incidence of PA was 0.4/100,000 population years in 1980 [3]. Furthermore, some hospital-based observational studies regarding the incidence of PA have been reported recently [[4], [5], [6], [7], [8]] in the era of the fourth generation CT.

In Japan, there were 111.5 CT scanners/1,000,000 population in 2017, which was the highest among Organization for Economic Co-operation and Development countries [9], whereas England, The Netherlands, and The USA had 9.5, 35.1, and 42.6 scanners/1,000,000 population. Thus, the liberal use of CT imaging along with an accessible medical system and universal public insurance system in Japan [10] could accurately reflect the true incidence of PA.

Therefore, the objective of this study was to evaluate the epidemiological and clinical features and outcomes of patients with PA in a prefectural-wide study in Japan.

## 2 Material and methods

### 2.1 Study design

Design: This was a multicenter retrospective cohort study.

Settings: There were 68 secondary and 3 tertiary care hospitals in the Miyagi prefecture. The population of the Miyagi prefecture was 2,344,062 as of April 1, 2011 [11]. Using medical records, we collected the clinical data of patients with PA who were admitted between April 2010 and March 2012. Data collection was completed in March 2017.

Participants: We enrolled adult patients with PA, spinal epidural abscess, pyogenic spondylitis, and pyogenic sacroiliitis to analyze spinal and perispinal infectious conditions. Finally, we selected patients with PA for analysis.

The inclusion criterion was diagnosis of PA using CT by staff radiologists at each hospital. The exclusion criterion was transfer to another hospital within the same prefecture for acute phase management.

### 2.2 Data collection

Data collection: Clinical background data including age, sex, body mass index (BMI), Charlson comorbidity index [12], and primary symptoms (fever and pain in the flank area) were collected. At the time of PA diagnosis, vital signs; time to diagnosis from onset of symptoms; presence of sepsis and septic shock; and laboratory data including the C-reactive protein level, white blood cell count, and platelet count and maximum abscess diameter on CT in the transaxial view were collected. Moreover, we recorded the causative pathogens, type of PA (primary or secondary), and the type of management (conservative or drainage, percutaneous or surgical). Clinical outcomes were analyzed using hospital mortality and hospital stay of survived patients as parameters.

### 2.3 Data definitions

Primary abscess was defined as PA due to the hematogenous spread of an infection from a distant site. If PA from a distant site was unknown and blood culture was positive, it was defined as primary PA. Secondary PA was defined as PA due to the direct expansion of adjacent infectious processes. Fever was defined as an axial temperature of 38.3 °C or higher [13] at the time of primary symptoms. Pathogens were identified as bacteria isolated from pus culture and/or two sets of blood cultures. Samples were considered to be contaminated if common skin bacteria (*Corynebacterium* spp.*, Bacillus* spp.*, Propionibacterium* spp.*, Viridans streptococci, Micrococcus* spp.*, and coagulase-negative Staphylococcus* spp.) were cultured in only one of the blood cultures [14].

Sepsis was defined as a systemic inflammatory response syndrome induced by infection. Septic shock was defined as a state of acute circulatory failure characterized by persistent arterial hypotension unexplained by other causes per the Sepsis-2 definition [15].

Conservative therapy was defined as intravenous antimicrobial use without drainage. CT-guided abscess drainage without open surgical procedures was defined as percutaneous drainage. If open surgical drainage was applied, cases were defined as “surgical” regardless of additional percutaneous drainage. The timing of the intervention was at the physician's discretion.

The maximum diameter of the abscess on CT was a single data set chosen from the largest abscess, even if there were bilateral PA.

### 2.4 Statistical analysis

Data are presented as median values (interquartile range) or n (%). If the field was missing, the value was excluded.

Previous studies have shown differences in clinical characteristics, treatments, and outcomes between patients with primary and secondary PA [2,16,17] as well as between patients with staphylococcal infection and other infections [1,2,18]. Therefore, we compared patients with primary and secondary PA and those with staphylococcal infection and other infections.

Categorical data were compared using the chi-square test or Fisher's exact test, as appropriate. Continuous data were compared using the Wilcoxon rank-sum test based on distributions.

Statistical analyses were conducted using JMP Pro. Ver. 14 (SAS institute Japan Ltd., Tokyo), and *p < 0.05* was considered significant.

This study was reported in line with the STROCSS guideline [19].

## 3 Results

### 3.1 1*. Characteristics and outcomes*

**Fig. 1:**
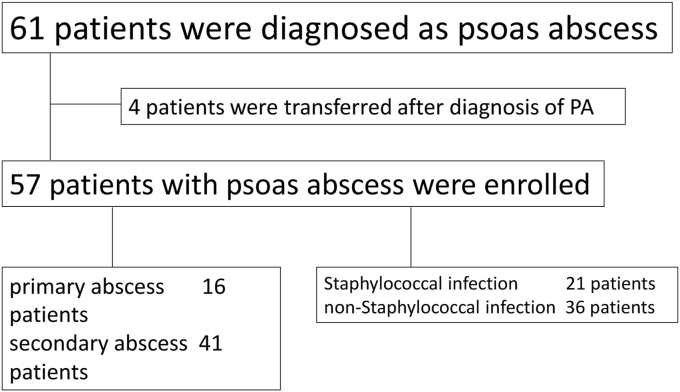
Flow diagram on the information obtained from the medical records of the study population.

Among the 71 hospitals, 68 (94%) participated in this study. There were 57 patients with adult PA during the study period from 9 hospitals (Fig. 1). The estimated incidence was 1.21 patients/100,000 population years during 2010–2012 (including all age groups).

The mean age was 72 years (range, 58–79 years), and 67% patients were male. The Charlson comorbidity index was 4 (3–6) points, and 47% of patients had diabetes. Fever and flank pain were observed as primary symptoms in 82% and 74% of patients, respectively. PA with both pain and fever as primary symptoms was observed in 34 cases (60%), and there were 2 cases (4%) without any symptoms. The mean duration to diagnosis from onset of symptoms ranged from 1 to 70 days. Ten patients (18%) had septic shock, and the hospital mortality rate was 12% (Table 1).

**Table 1:** Patient characteristics of psoas abscess (n = 57).

	Total (n = 57)	Primary (n = 16)	Secondary (n = 41)	*p-value*
Age	72 (58–79)	75 (62–80)	68 (58–78)	0.45
Male, n (%)	38, 67%	9, 56%	29, 71%	0.36
BMI	19.4 (21.5–24.9)	20.8 (18.6–23.8)	22.0 (19.5–25.0)	0.34
Charlson comorbidity index	4 (3–6)	5 (4–7)	4 (3–6)	0.44
Diabetes, n (%)	27, 47%	7, 44%	20, 49%	0.78
Primary symptom: Pain	42, 74%	8, 50%	34, 83%	***0.02***
Primary symptom: Fever	47, 82%	15, 94%	32, 78%	0.25
Time to diagnosis from primary symptoms (days)	8 (4–18)	10 (2–21)	8 (4–15)	0.48
Clinical severity on admission				
Sepsis	47, 82%	15, 94%	32, 78%	0.25
Septic shock	10, 18%	4, 25%	6, 15%	0.44
C-Reactive Protein (mg/dL)	17.0 (9.4–26.0)	14.9 (7.0–35.1)	17.1 (11.7–25.2)	0.72
White Blood Cell ( × 10^2^/μL)	113 (75–170)	118 (71–188)	112 (82–152)	0.22
Platelet ( × 10^3^/μL)	29.4 (12.9–49.1)	20.2 (11.6–46.1)	29.4 (14.0–56.9)	0.25
Abscess information				
Maximum diameter (mm)	32 (20–42)	25 (20–52)	32 (19–40)	0.17
Bilateral muscle abscess	14, 25%	3, 19%	11, 26%	0.52
Epidural abscess	18, 32%	0, 0%	18, 44%	0.02
Other abscesses	5, 8.8%	1, 6%	4, 10%	0.66
Therapy				
Conservative	32, 56%	8, 50%	24, 59%	0.56
percutaneous drainage	17, 30%	6, 38%	11, 27%	0.43
surgical drainage	8, 14%	2, 12%	6, 14%	0.52
Intervention for primary disease*	5, 8.8%-	0, 0%	5, 12%	–
Microbiology				
MSSA	15, 26%	4, 25%	11, 27%	1.00
MRSA	6, 11%	3, 19%	3, 7%	0.33
*E. coli*	5, 9%	1, 6%	4, 10%	0.66
Unknown	22, 39%	6, 38%	16, 39%	0.92
Clinical outcome				
Hospital mortality	7, 12%	4, 25%	3, 7%	***0.04***
Hospital stay of survived patients (days)	54 (26–78)	60 (43–100)	49 (25–75)	0.81

BMI, body mass index; MSSA, methicillin-sensitive *Staphylococcus aureus*; MRSA, methicillin-resistant *Staphylococcus aureus*.

### 3.2 Comparison between primary and secondary PA

Primary abscess occurred in 16 patients (28%), and infectious endocarditis was observed in 1 patient (1.8%). Secondary abscess occurred in 41 patients (72%), and the most common origin of PA was bony infection (Table 2). Of the patients with secondary PA, 44% had an epidural abscess. There were 28 patients with pyogenic spondylitis with PA, and 8 patients underwent laminectomy.

**Table 2:** Disease origin of secondary psoas abscess.

Disease origin	n, %
Pyogenic spondylitis	28, 68%
Pyogenic sacroiliitis	4, 9.8%
Urinary tract infection	6, 14.6%
Vascular infection	2, 4.8%
Gastrointestinal tract infection	1, 2.4%

The primary symptoms of pain (50% vs. 83%, *p* = *0.02*), epidural abscess (0% vs. 44%, *p* = *0.02*), and hospital mortality (25% vs. 7%, *p* = *0.04*) were different between patients with primary and secondary abscesses.

### 3.3 Comparison between staphylococcal infection and other infections

The most common pathogen was *Staphylococcus aureus* (37%), and 6 cases (11%) were caused by *methicillin-resistant S. aureus*. Other pathogens included *Pseudomonas aeruginosa*, *Enterococcus faecalis*, *Streptococcus anginosus*, *Streptococcus agalactiae*, *Klebsiella pneumoniae*, group A *Streptococcus*, *Serratia marcescens*, *Streptococcus constellatus*, *Staphylococcus epidermidis*, and unknown pathogens. Age (61 vs. 76 years, *p* = *0.04*), septic shock (33% vs. 8.3%, *p* = *0.03*), bilateral PA (57% vs. 86%, *p* = *0.02*), and epidural abscess (52% vs. 19%, *p* = *0.02*) were different between *S. aureus* infections and others (Table 3). There was no difference in hospital mortality between the groups (9.5 vs. 13.9%, *p* = *0.62*).

**Table 3:** Clinical characteristics between Staphylococcal infection and non-Staphylococcal infection.

Pathogen	*S. aureus* (n = 21)	Others (n = 36)	*p-value*
Age	61 (53–76)	76 (64–80)	***0.04***
Male, n (%)	14, 67%	24, 67%	1.00
Body mass index	20.3 (18.0–23,1)	22.2 (20.2–27.8)	***0.04***
Charlson comorbidity index	4 (3–6)	5 (3–7)	0.33
Diabetes	10, 48%	17, 47%	1.00
Sepsis	20, 95%	27, 75%	0.08
Septic shock	7, 33%	3, 8.3%	***0.03***
Primary abscess	7, 33%	9, 25%	0.55
Bilateral muscle abscess	12, 57%	31, 86%	***0.02***
Epidural abscess	11, 52%	7, 19%	***0.02***
Hospital mortality	2, 9.5%	5, 13.9%	0.62
Hospital stay of survived patients	71 (29–86)	49 (23–62)	0.73

## 4 Discussion

The estimated prevalence of PA was 1.21 patients/100,000 population years in the Miyagi prefecture between 2010 and 2012. This is the first report of district-based epidemiology of PA in Japan. In addition, the results showed that secondary PA with skeletal pathology accounted for most cases and was accompanied by epidural abscess. *S. aureus* was the most common causative bacterium with a higher complication rate of septic shock than other bacteria. The hospital mortality rate was 12%, which was similar to a previous report [1].

In previous reports, primary PA was reported to develop most likely into secondary to staphylococcal bacteremia from an occult infection in the body owing to the rich vascular supply of the psoas muscle in younger patients in developing and tropical countries (Asia and Africa). Contrarily, secondary PA with enteric origin is commonly found in Europe and North America, and mixed enteric bacteria are the major pathogens involved [20]. Primary and secondary PAs occurred at a frequency ratio of 11–89%. Secondary PA accounted for 72% in this study. Thus, the ratio of primary and secondary PA did not differ from previous reports, and the proportion of secondary PA did not increase in Japan despite the liberal use of CT imaging.

Bony infection accounted for most secondary PA cases in this study. With time, the origin of secondary PA has changed from tuberculosis [21] and digestive disease [22] to skeletal pathology [5,[23], [24], [25]]. The results of the present study are consistent with these findings. Because PA is often diagnosed prior to detection of the primary disease, clinicians must be careful to avoid overlooking these lesions. Pyogenic spondylitis commonly arises from the hematogenous spread of bacteria, and the most common pathogen is *S. aureus* [26]. This might reflect the homology of characteristics between primary and secondary PA.

In this study, the most common pathogen for both primary and secondary PA was *S. aureus* that leads to additional complications such as septic shock, epidural abscess at a younger age, and single-sided abscess more frequently than other pathogens. *S. aureus* was the predominant primary cause of PA [27], which is consistent with our findings. Moreover, *S. aureus* has been reported as a major pathogen of pyogenic spondylitis [28]. In a previous study, however, the clinical presentation had an insidious onset with vague nonspecific features, despite the same pathogen of *S. aureus* [29]. In this study, almost 40% of pathogens were unknown. In a previous report, pathogens were not identified in 25% of cases [2].

The limitations of this study are as follows. Owing to the retrospective nature of the study, we were unable to measure all the factors. Moreover, there is a possibility that cases were missed as PA was not diagnosed or described in the medical records or because the patient was treated conservatively.

## 5 Conclusions

In the Miyagi prefecture of Japan, the estimated prevalence of PA was 1.21/100,000 population years and the hospital mortality rate was 12%. Secondary PA consisted of more than 70% of cases, and *S. aureus* was the most common causative bacteria with a higher complication rate of septic shock than other bacteria. Future prospective studies are needed to clarify the epidemiology of PA and improve patient outcomes.

## Ethical approval

Ethical approvals were obtained by Tohoku University Hospital ethical committee (2012-1-30), and each participant hospitals followed to this approval.

## Funding

None.

## Author contribution

Takeaki Sato: Conceptualization, Methodology, Investigation, Writing-original draft preparation. Daisuke Kudo: Formal analysis. Shigeki Kushimoto Supervision.

## Research Registration Unique Identifying Number (UIN)

Name of the registry: Research Registry

Unique Identifying number or registration ID: Researchregistry6371

Hyperlink to your specific registration (must be publicly accessible and will be checked): https://www.researchregistry.com/browse-the-registry#home/registrationdetails/5fdb1ab244899d001ded0200/

## Guarantor

Takeaki Sato is a guarantor.

## Provenance and peer review

Not commissioned, externally peer-reviewed.

## Data statement

All the data were presented in the manuscript.

## Declaration of competing interest

None.
